# Exploring the Influence of Chemical Conditions on Nanoparticle Graphene Oxide Adsorption onto Clay Minerals

**DOI:** 10.3390/molecules28166162

**Published:** 2023-08-21

**Authors:** Marwa I. M. Ibrahim, Elsayed A. M. Awad, Salah M. M. Dahdouh, Wafaa M. T. El-Etr, Samy A. Marey, Ashraf Atef Hatamleh, Mohsin Mahmood, Ahmed S. Elrys

**Affiliations:** 1 Department of Soil Science, Faculty of Agriculture, Zagazig University, Zagazig 44511, Egypt; 2 Department of Soil Physics and Chemistry, Soil, Water and Environment Research Institute (SWERI), The Agricultural Research Center (ARC), Giza 12619, Egypt; 3 King Saud University, Riyadh 11451, Saudi Arabia; samarey@ksu.edu.sa; 4 Department of Botany and Microbiology, College of Science, King Saud University, P.O. Box 2455, Riyadh 11451, Saudi Arabia; ahatamleh@ksu.edu.sa; 5 Center for Eco-Environment Restoration Engineering of Hainan Province, College of Ecology and Environment, Hainan University, Haikou 570228, China; 6 Liebig Centre for Agroecology and Climate Impact Research, Justus Liebig University, 35390 Giessen, Germany

**Keywords:** adsorption, bentonite, graphene oxide, ionic strength, kaolinite, pH

## Abstract

High concentrations of graphene oxide (GO), a nanoparticle substance with rapid manufacturing development, have the ability to penetrate the soil surface down to the mineral-rich subsurface layers. The destiny and distribution of such an unusual sort of nanomaterial in the environment must therefore be fully understood. However, the way the chemistry of solutions impacts GO nanoparticle adsorption on clay minerals is still unclear. Here, the adsorption of GO on clay minerals (e.g., bentonite and kaolinite) was tested under various chemical conditions (e.g., GO concentration, soil pH, and cation valence). Non-linear Langmuir and Freundlich models have been applied to describe the adsorption isotherm by comparing the amount of adsorbed GO nanoparticle to the concentration at the equilibrium of the solution. Our results showed fondness for GO in bentonite and kaolinite under similar conditions, but the GO nanoparticle adsorption with bentonite was superior to kaolinite, mainly due to its higher surface area and surface charge. We also found that increasing the ionic strength and decreasing the pH increased the adsorption of GO nanoparticles to bentonite and kaolinite, mainly due to the interaction between these clay minerals and GO nanoparticles’ surface oxygen functional groups. Experimental data fit well to the non-linear pseudo-second-order kinetic model of Freundlich. The model of the Freundlich isotherm was more fitting at a lower pH and higher ionic strength in the bentonite soil while the lowest R^2^ value of the Freundlich model was recorded at a higher pH and lower ionic strength in the kaolinite soil. These results improve our understanding of GO behavior in soils by revealing environmental factors influencing GO nanoparticle movement and transmission towards groundwater.

## 1. Introduction

Graphene is a two-dimensional carbon-based nanomaterial with exceptional physiochemical properties, including major-specific area and large electric and thermal conductivity [1,2]. As a result, it is used in many different disciplines and applications [3]. Graphene oxide (GO) is one of the most extensively utilized forms of graphene, and it is employed in many applications, including electronics, energy devices, biosensors, biomedicals, supercapacitors, membranes, catalysts, and water purification. It also has many possible applications, including agricultural, biological, and environmental protection [4]. Due to the increasing manufacture and use of GO, it is expected to be released into the environment, negatively affecting the ecosystem functions. Hence, a large amount of research has been done on the movement and fate of GO material in the environment. After oxidation, carboxylic groups dominate the GO sheet’s edges, whereas hydroxyl and epoxied groups predominate in the basal plane [4,5,6]. Some investigations have also found sulfur-containing functional groups in GO as a result of sulfuric acid (H_2_SO_4_) impurities present during production [7]. Furthermore, GO has a high oxygen concentration, acts as an insulator, and is highly hydrophilic. Under normal conditions, GO is a negatively charged material because of its numerous surface O-functional groups. Ref. [8] added that GO has high mobility in porous media. The GO also has outstanding adsorbent capabilities, especially in solid–liquid systems, due to the scale of the generated surface area, the number of groups at the surface that contain oxygen, and the good dispersion characteristics [7,9]. Environmental quality studies are focusing on the properties of GO in soil and sediment systems. This may be due to the interaction between GO and solid material, which have a substantial impact on GO transport in natural subsurface environments [10,11]. Previous investigations showed that GO nanoparticles might be very stable in environmental circumstances. Nanoparticle hetero-aggregation with clay minerals is a critical step for nanoparticle constancy [12,13]. The surface shape and charge of clay particles, for example, influence the stability of GO nanoparticles according to hetero-aggregation. Thus, the interaction of GO nanoparticles with clay minerals is probably important for GO nanoparticle transit and detention in porous settings, which is still only tentatively known.

Chemical and mechanical stabilities, layered structure, tendency to hold water in the interlayer sites, and high specific surface area and cation exchange capacity (CEC) have made clay minerals excellent adsorbent materials [14]. Among clay minerals, bentonite, which is very soft plastic clay and is a 2:1 clay mineral type composed primarily of montmorillonite, is commonly used in many fields, including painting, foundry, and ceramics [14]. It can also be applied in wastewater treatment by means of adsorption. Kaolinite, a 1:1 clay mineral, is also a common clay mineral in subsurface areas and is used as a good adsorption medium in previous studies [15,16]. For example, it was used as an alternative adsorbent by [17,18] to remove minerals and dyes from wastewater due to its low cost and high adsorption performance. Depending on the pH of the solution, kaolinite was used as an active adsorption site for eliminating hazardous compounds from wastewater [19,20]. Additionally, Ref. [21] mentioned that montmorillonite and kaolinite have the strongest affinities for GO. For instance, the existence of kaolinite and montmorillonite inhibited GO transport to varying degrees [22]. The spots with a positive charge on clay margins (which served as ideal deposition sites) mostly hampered transport. Because of its large proportion of edge area, kaolinite displayed the most essential transport-inhibition influences.

The influence of pH and ionic strength (IS)-dependent GO adsorption on montmorillonite and kaolinite was studied by Sotirelis and Chrysikopoulos [15], who found that the chemical composition and structural characteristics of these minerals differed in GO adsorption. The different montmorillonite layers have regular negative charges due to isomorphic replacements, and pH-dependent (positive or negative) charges could be found at the margins [23]. Because of this, negatively charged nanoparticles such as GO can benefit from the favorable adsorption surfaces offered by montmorillonite colloids, allowing for more control over GO’s environmental fate. Chrysikopoulos et al. [24] indicated that GO nanoparticle retention enhanced when decreasing the pH of the solution by boosting hetero-aggregation between GO nanoparticles, which was in line with other findings [25]. Furthermore, ref. [26,27] spotted that the influence of IS is generally compatible with the principles driving the transport of nanoparticles that are negatively charged [28]. Increasing IS compacts the double-layer and lowers the double-layer repulsion between grain surfaces and nanoparticles [29]. At a higher IS level, heteroaggregation rates increased dramatically due to a decrease in electrical double-layer repulsion. However, ref. [30] mentioned that an increasing sodium (Na^+^) concentration reduced the double layer’s thickness, this might result in electrical protection for the nanoparticle colloids. In addition, GO nanoparticles were better electrostatically attached to clay minerals when ionic strength was increased or the pH was decreased. The amount of GO particles discovered to be linked to clay mineral particles increased as calcium (Ca^2+^) concentration increased. This occurred due to the interaction among the clay minerals and the GO nanoparticles’ surface oxygen functional groups. Ref. [27] found that Ca^2+^, at 0.5 mM, significantly hampered the transport of GO in the soil. This was attributed to the possibility that Ca^2+^ could promote GO aggregation and bond construction between soil grains and GO through their respective surfaces’ O-functionalities. However, as compared to Na^+^, GO had a larger enhancing influence on the transportation of mineral colloid interactions with Ca^2+^. Moreover, GO adsorption increased with increasing its concentration and contact duration but decreased with increasing adsorbent dosage [31]. However, the way the chemistry of solutions (GO concentrations, pH, and IS [Na^+^ and/or Ca^2+^]) impacts GO nanoparticle adsorption on clay minerals is still unclear.

The pseudo-second-order kinetic model [32] offered the best explanation for the kinetic data of the adsorption process. The thermodynamic investigation proved the exothermic nature of the adsorption process. In kinetic batch testing, the attachment of GO nanoparticles to kaolinite colloids was seen to follow a pseudo-second-order model [24]. The results of the kinetic analysis also showed that the pseudo-second-order kinetic model successfully explained the attachment of GO to the surface of montmorillonite. The GO adsorption to montmorillonite happened quite quickly, reaching equilibrium in less than 30 min [33]. The GO attachment to montmorillonite was well described by the Freundlich isotherm equation. Moreover, some of the thermodynamic properties associated with GO adsorption onto clay minerals include the maximum adsorption amounts, Gibbs free energy change, enthalpy change, and entropy change [31,34]. These thermodynamic properties can be affected by electrostatic contacts, van der Waals forces, hydrophobic interactions, and the molecule cross-sectional area of the adsorbate. A study by [35] revealed that the pH-dependent adsorption of gallic acid onto Na-montmorillonite clay includes electrostatic contact, Van der Waals forces, and coordinative bonding. Overall, depending on the specific adsorbate and clay mineral involved, the Gibbs free energy, enthalpy, and entropy changes related to GO adsorption onto clay minerals can vary.

Finally, with the increasing mass manufacturing and use of GO, there is a greater risk of release into underground environments, where clay mineral particles are abundant. Therefore, understanding the interaction of GO and clay minerals is critical to determine the fate and behavior of GO in natural soil–water systems. Thus, the current work was planned to investigate the adsorption GO nanoparticle and its dynamics on two clay minerals (bentonite and kaolinite) under different chemical conditions. Batch examinations were carried out in the laboratory under various solution chemistry circumstances, such as varying GO nanoparticle concentrations, pH, and IS (Na^+^ and/or Ca^2+^). The results of this study are critical for analyzing the potential risks of GO release into the environment.

## 2. Results and Discussion

In this study, we determined how the GO nanoparticle adsorption rate changed in response to various environmental factors. These settings included varying quantities of GO in the solution with clay minerals (bentonite and kaolinite) under different environmental conditions (IS and pH). The kinetic studies were carried out by evaluating both K_F_ (adsorptive capacity) and n (adsorption intensity). The GO adsorption responses to the equilibrium ion concentration in the relevant solution were also assessed. This work aids in our understanding of the fate and behavior of GO nanoparticles in soils as well as the degree to which environmental conditions influence GO movement in the soil.

### 2.1. Influence of Concentrations on GO Nanoparticle Adsorption

**Figure 1:**
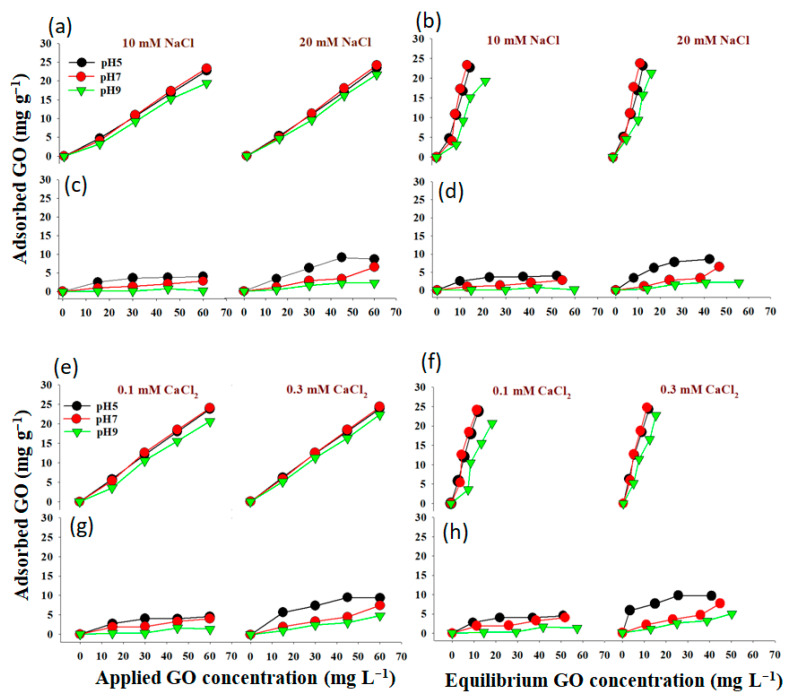
Influence of applied and equilibrium GO amounts in the relevant solution on absorbed GO onto bentonite (**a**,**b**,**e**,**f**) and kaolinite (**c**,**d**,**g**,**h**) under different pH and ionic strength (NaCl and CaCl_2_).

The GO behavior was investigated by evaluating the reactions of the quantity of adsorption under different IS (NaCl or CaCl_2_) and pH conditions by evaluating the applied and equilibrium concentrations (Figure 1). The relationship between the concentration of applied and equilibrium GO and the quantity of GO adsorbed onto various clay minerals (bentonite and kaolinite) was also evaluated (Figure 1). Our results showed that the amount of adsorption into the soil improved with an increasing applied GO concentration, which was a positive trend [33]. Recently, ref. [36] also reported that the adsorption capacity of clay mineral was enhanced with increasing GO concentration, which was in line with our findings. Furthermore, ref. [37] showed that higher GO concentration stimulated the GO adsorption capacity on clay minerals. It appears that higher GO concentrations were generally more efficient. When the equilibrium GO concentration was raised (Figure 1), the quantity of adsorbed GO increased, especially when bentonite was used (Figure 1a,c). A higher amount of adsorption was obtained with bentonite at a GO concentration of 60 mg L^−1^ GO, pH of 5, and IS of 20 mM NaCl (Figure 1a). When treating with high concentrations of GO, in contrast, the amount of GO adsorbed on kaolinite was slightly less than that of bentonite (Figure 1). This result may indicate that in the experimental circumstances (pH 5.0 and 20 mM NaCl), GO and clay minerals were both negatively charged [21,38]. The negatively charged clay surface can repel negatively charged GO. As a result, electrostatic interaction may be responsible for GO adsorption on clay minerals [21].

The relationship between the quantity of GO adsorbed onto clay minerals and the concentration of GO either applied or at equilibrium under different IS-CaCl_2_ was shown in Figure 1e–h. Increases in the amount of GO adsorption onto both clay minerals with increasing GO concentrations either applied or at equilibrium were recorded, indicating that higher GO concentrations were typically more effective. Bentonite with 0.3 mM CaCl_2_ and pH 5 had the greatest adsorption (Figure 1e,f). This was different from kaolinite soil as higher concentrations of GO led to a slight decrease in the amount of adsorbed GO compared to the lower concentrations under the same experimental conditions. The slight rise in adsorbed GO was likely due to intense competition from other anions for accessible adsorption sites in the soils [39]. After the equilibrium GO concentration reached an assured level, the fast increase in adsorbed GO indicated that the GO ion exchanged some of the exchangeable anions with an increase in soluble GO concentration [21]. Then, as the equilibrium GO concentration increased, the rate of increase of adsorbed GO decreased again; this could be attributed to the anion exchange site saturation approach.

According to the findings, various pathways were implicated in the enhancing effect of GO when Ca^2+^ was present. It is now widely acknowledged that O-functional groups (e.g., hydroxyl) exist in significant quantities on the surfaces of GO and clay minerals. Understanding the mechanisms underlying the co-transport of GO nanoparticles and colloids in the presence of Ca^2+^ would be helpful [40]. When the effects consisted of divalent cations (i.e., Ca^2+^), the breakthrough curves and retention patterns of GO and clay minerals are illustrated. The results were very comparable to those of Na^+^. In other words, whereas colloids may impair GO transport, GO facilitates it, and this improvement becomes more important as the GO concentration rises. As already indicated, these findings were attributed to electrostatic interaction and GO–clay mineral hetero-aggregation [15]. Additionally, the straining impact was greater in the case of Ca^2+^ compared to Na^+^. However, when compared to Na^+^, GO displayed a larger enhancing effect on GO adsorption in the presence of Ca^2+^ [41]. Moreover, refs. [42,43,44] showed that due to their cation-bridging properties, divalent cations usually have a higher influence on the interaction between minerals and nanomaterials. As a result, in the presence of Ca^2+^, more GO nanoparticles were adsorbed on clay minerals than Na^+^.

### 2.2. Influence of Clay Minerals on GO Nanoparticles Adsorption

**Figure 2:**
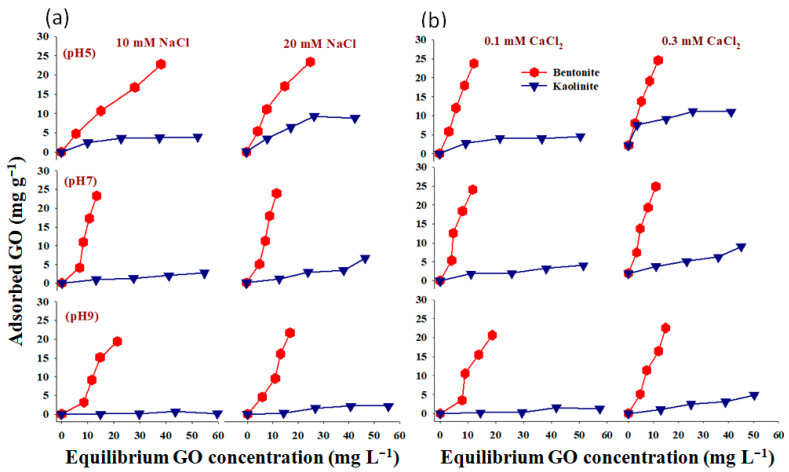
Influence of bentonite and kaolinite on GO nanoparticle adsorption under different pH and ionic strength (NaCl (**a**) and CaCl_2_ (**b**)).

The adsorption of GO nanoparticles on different clay minerals (bentonite and kaolinite) was investigated and explained under different experimental conditions (Figure 2). Our results indicated that both the GO nanomaterial and clay particles were negatively charged and that GO adsorption with bentonite was superior to kaolinite (Figure 2a,b). Furthermore, the effects of GO concentration on its adsorption showed the optimal experimental settings (pH 5.0 and IS 20 mM NaCl and/or 0.3 CaCl_2_). The negatively charged GO has been rejected by the negatively charged clay surface [38]. As a result, the electrostatic link could be attributed to GO adsorption on clay minerals. Furthermore, the hydrophobic influence on the bonding GO-clay minerals would be negligible. Additionally, according to [33,45], bentonite is a bulge smectite clay with constant negative charges on the basal planes as a result of an isomorphic substitution of silicon (Si) and aluminum (Al) ions in its structure as well as subjunctive charges on amphoteric edge sites (primarily, Al-OH and Si-OH). According to [46], the surface irregularity and angularity, chemical contaminants, and charge on the inhomogeneous surface of minerals, which are known to produce localized zones of favorable interaction, aid in adhesion even in difficult conditions [47]. Under the same solution chemistry conditions, the rise in GO affinity was on the order of bentonite > kaolinite (pH 5.0, 20 mM NaCl, and/or 0.3 mM CaCl_2_). The degree of clay bulge is related to the amount of nanoparticles connected to the clay particles. The structure of bentonite allows for greater swelling than kaolinite [48]; thus, more GO is attached to the bentonite particles [49].

### 2.3. Influence of pH on GO Nanoparticles Adsorption

**Figure 3:**
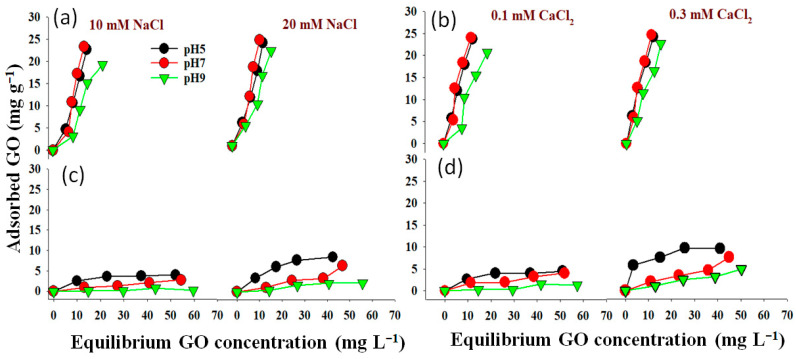
Influence of pH on the adsorption of GO nanoparticles on bentonite (**a**,**b**) and kaolinite (**c**,**d**) under different ionic strength (IS) conditions.

The influence of pH on Go nanoparticle adsorption onto bentonite and kaolinite under different IS levels (10 and 20 mM NaCl and/or 0.1 and 0.3 mM CaCl_2_) was shown in Figure 3. Positive responses were observed at pH 5 compared to pH 7 or 9. The decrease in GO nanoparticle mass seen on clay minerals with higher pH values is explained by the electrostatic forces and the crystalline makeup of the clay mineral. Furthermore, higher IS (20 mM NaCl or 0.3 mM CaCl_2_) has often been more effective than the lowest IS (10 mM NaCl or 0.1 mM CaCl_2_). The current study also found that more GO nanoparticles were adsorbed to bentonite than kaolinite at all examined pH levels (Figure 3). Ref. [50] reported that at higher pH levels, negative charges are present on the Si-O and Al-O faces of bentonite and kaolinite, respectively, resulting in lower interactions between clay particles and GO nanoparticles. However, ref. [27] noticed that increasing pH within the test pH range of 4 to 9 resulted in improved GO nanoparticle transport in the soil, but the influences were only marginal. Since pH had little impact on the Z potential of GO nanoparticles, its impacts on soil particles were mostly responsible for the minor transport-enhancement effects that were found. As the pH rises, the surface charges of clay minerals become increasingly negative [51]. More importantly, pH can influence the movement of nanoparticles under unfavorable depositions, such as metal oxides, by hiding the heterogeneities of grain surfaces [52,53]. The surfaces of some soil minerals, such as Al_2_O_3_ and Fe_2_O_3_, may be positively charged at an acidic pH, and a rising pH could remove and/or reverse these positive surface sites. Refs. [33,54] pointed out that as the pH drops, the carboxyl groups, which are likely found at the margins of the GO nanosheets, become more protonated. This causes GO nanosheets to become less hydrophilic [55].

### 2.4. The Relationship between Ionic Strength and GO Nanoparticle Adsorption

**Figure 4:**
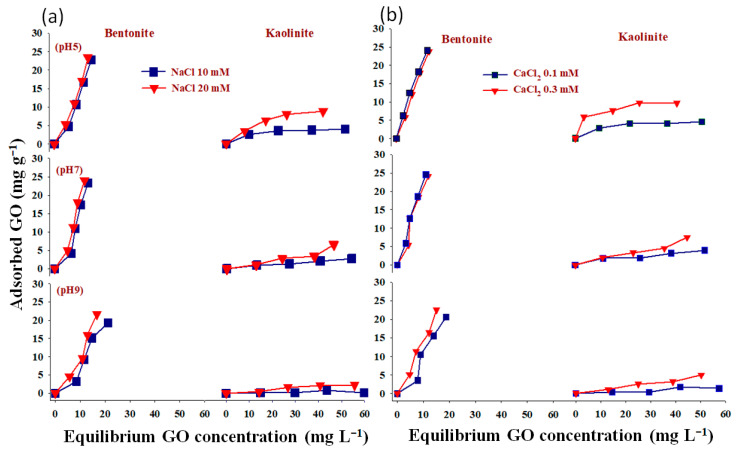
The relationship between IS-NaCl (**a**) and IS-CaCl_2_ (**b**) GO nanoparticle adsorption onto bentonite and kaolinite.

The relationship between the amount of GO nanoparticle adsorption onto bentonite and kaolinite under various levels of IS (10 and 20 mM NaCl and/or 0.1 and 0.3 mM CaCl_2_) is shown in Figure 4. As IS concentration varied from 10 to 20 mM NaCl at pH 5, a positive trend was frequently observed. When using 0.3 mM CaCl_2_ instead of 0.1 mM CaCl_2_, a similar trend was noticed. Our results also confirmed that bentonite clay minerals were more effective for GO adsorption than kaolinite under the same conditions. These findings were in line with those of [27], who pointed out that while the influent’s IS elevated from 0 to 10 mM NaCl, a significant retention of GO was only recorded at 25 mM NaCl and above. In addition, ref. [21] reported that as the Ca^2+^ concentration increases, more GO gets linked to clay particles. Strong GO–clay bridging effects are theoretically encouraged by the presence of Ca^2+^. As previously stated, the surface O-functionalities, carboxyl, carbonyl, hydroxyl, and phenol, are especially plentiful in GO. As the Ca^2+^ concentration rises, more Ca^2+^ may create complexes by acting as a bridge between the surface functional groups of clay minerals and GO nanoparticles. As a result, more montmorillonite clay material particles are joined with GO nanoparticles.

### 2.5. Adsorption Isotherms

**Figure 5:**
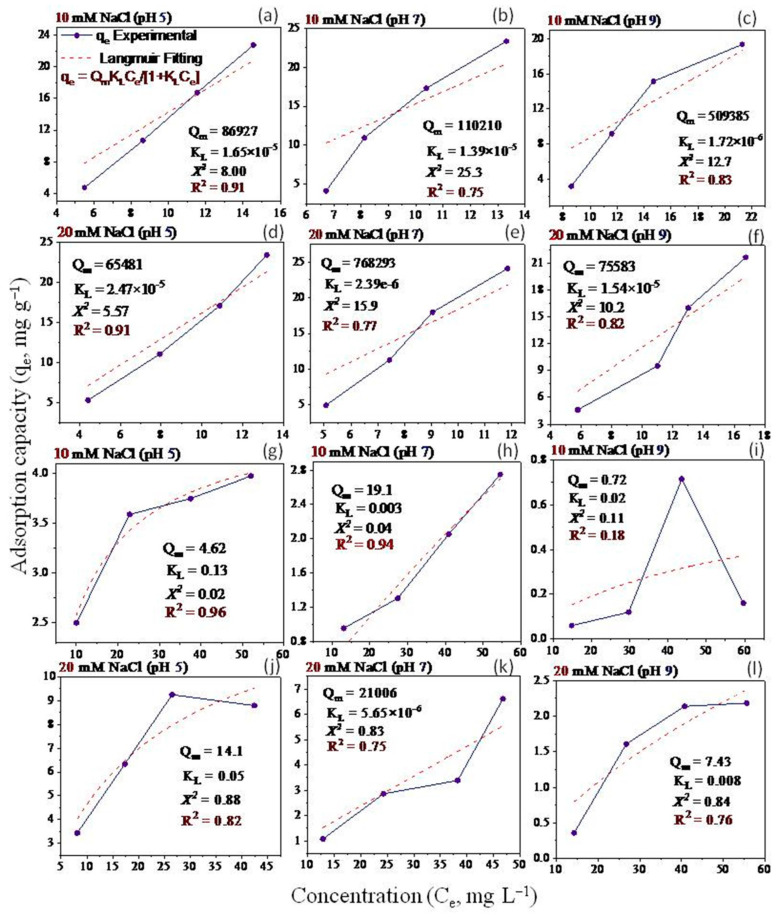
Langmuir isotherm for GO equilibrium adsorption onto bentonite (**a**–**f**) and kaolinite (**g**–**l**) under different experimental conditions of pH and IS-NaCl.

**Figure 6:**
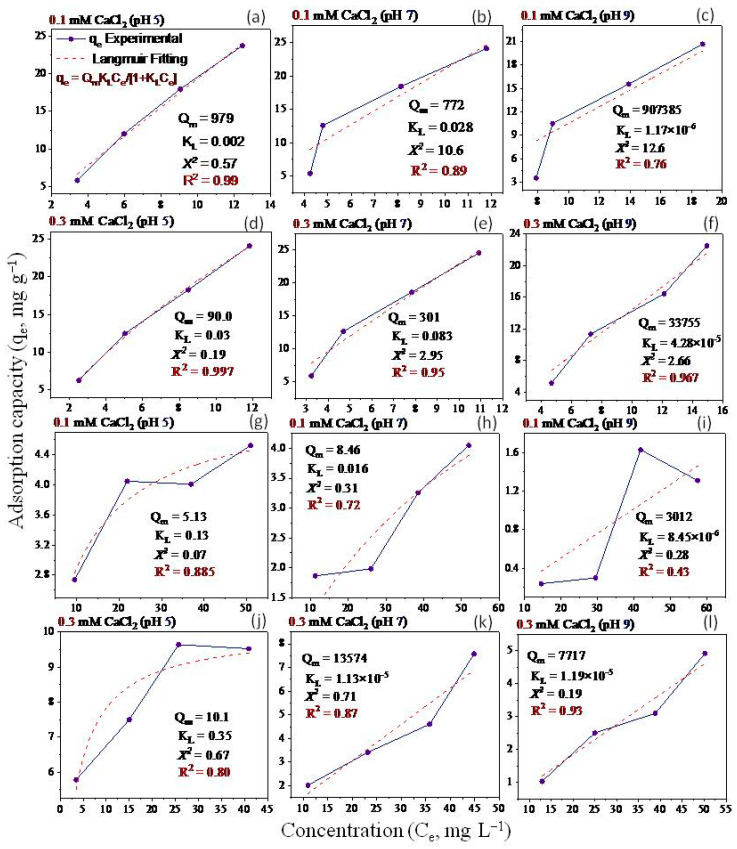
Langmuir isotherm for GO equilibrium adsorption onto bentonite (**a**–**f**) and kaolinite (**g**–**l**) under different experimental conditions of pH and IS-CaCl_2_.

**Figure 7:**
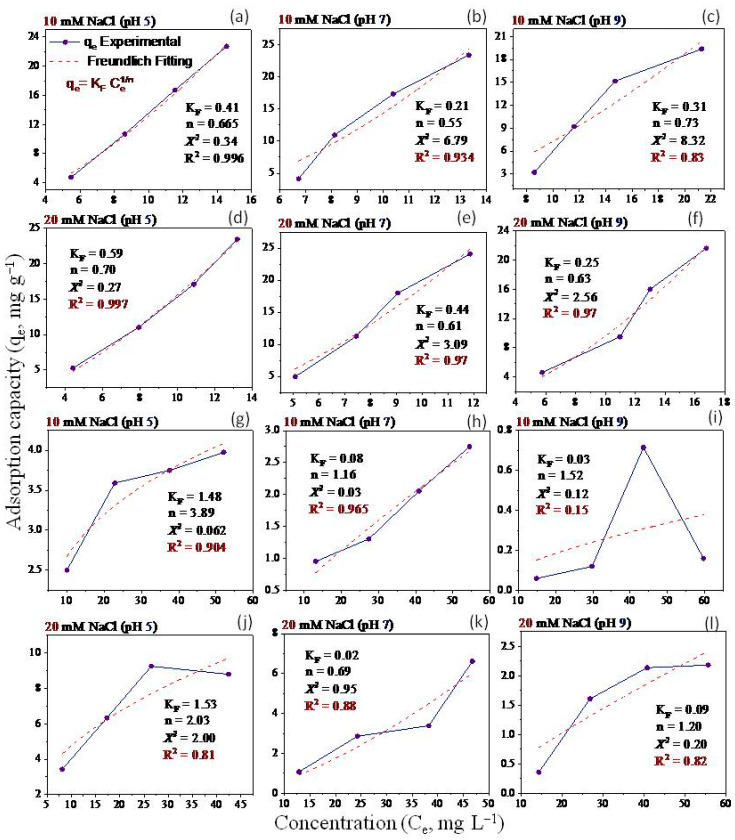
Freundlich isotherm for GO equilibrium adsorption onto bentonite (**a**–**f**) and kaolinite (**g**–**l**) under different experimental conditions of pH and IS-NaCl.

**Figure 8:**
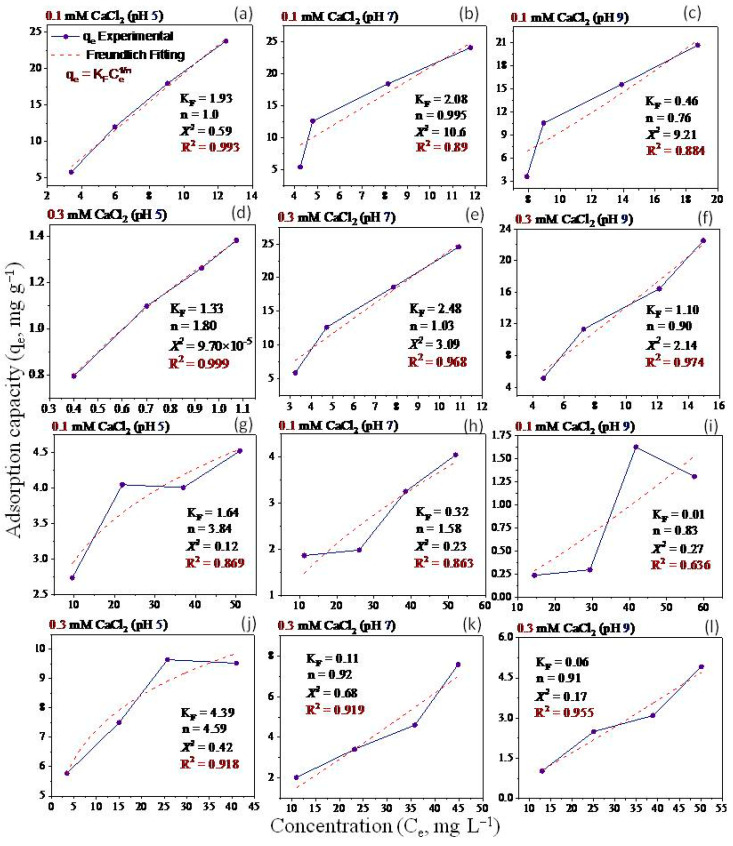
Freundlich isotherm for GO equilibrium adsorption onto bentonite (**a**–**f**) and kaolinite (**g**–**l**) under different experimental conditions of pH and IS-CaCl_2_.

Non-linear Langmuir and Freundlich models have been applied to fit the equilibrium data (Figure 5, Figure 6, Figure 7 and Figure 8). Both models were utilized to describe GO adsorption on clay minerals under various experimental settings. These models described the adsorption isotherm by comparing the amount of adsorbed GO nanomaterial to the concentration at equilibrium of the solution. The goodness of fit for a certain model was established by looking at the R^2^ and the Chi-square values, according to [56]. The Langmuir and Freundlich coefficient and the Chi-square for non-linearized equations were obtained by plotting graphs between q_e_ vs. Ce using experimental and predicted values from a non-linear model. Our analysis revealed that the adsorption of GO nanoparticles on the studied clay minerals followed the non-linear Freundlich model rather than the non-linear Langmuir model according to the R^2^ and *x*^2^ values (Figure 5, Figure 6, Figure 7 and Figure 8). Thus, the model of the Freundlich isotherm fits the tested data well under static and dynamic settings in the vast majority of the cases examined in this study, which was in harmony with other adsorption studies [57,58]. However, this pattern differed under different chemical conditions. For example, the model of the Freundlich isotherm was more fit at a low pH than at a medium and higher pH when the bentonite soil was used. The same trend was also true under high IS levels (20 mM NaCl or 0.3 CaCl_2_) compared to the low IS levels. The lowest R^2^ value (0.18 and 0.64) of the Freundlich model was recorded at the higher pH under the low levels of NaCl (10 mM) and CaCl_2_ (0.1 mM) when kaolinite soil was used.

The adsorption intensity (n) was also used to identify the best-fitting non-linearized Freundlich isotherm mode. The adsorption intensity values of 2–10, 1–2, and <1 represent good, moderate, and poor adsorption, respectively [59]. When bentonite soil was tested, our results showed that the adsorption intensity was moderate under both concentrations of CaCl_2_ (0.1 and 0.3 mM) but at low and moderate pH only (Figure 8a,b,d,e). However, the adsorption intensity was poor at pH 9 under both CaCl_2_ concentrations (Figure 8c,f). For the kaolinite soil, the adsorption intensity was good at both concentrations of CaCl_2_ (0.1 and 0.3 mM) but in acidic conditions (pH 5) only (Figure 8g,j). Refs. [60,61] illustrated that the exposed hydroxyl-terminated planes of a crystalline structure and fractured edges of the clay minerals both have a significant concentration of amphoteric sites (such as octahedral Al-OH sites). At pH 5, the Al-O face or edge of clay minerals was positively charged, and thus, more GO nanoparticles with negative charges are supposed to be drawn to the Al-O face or edge. A moderate adsorption intensity was also recorded at the low CaCl_2_ concentration (0.1 mM) when pH was 7 (Figure 8h). In contrast, poor adsorption was noted for both NaCl concentrations (10 and 20 mM) at all pH levels in the bentonite soil (Figure 7a–f). However, in the kaolinite soil, good adsorption was observed at both NaCl levels when pH was 5 (Figure 7g,j). We also found that adsorption intensity in the kaolinite soil was moderate under the low concentration of NaCl (10 mM) at pH 7 and 9 (Figure 7h,i) and also under high concentration of NaCl but at pH 9 only (Figure 7l). These results suggested that even in unfavorable circumstances, adsorption may occur due to the roughness of the surface, the angularity of the mineral, chemical impurities, and the surface charge heterogeneity of the minerals [47]. These factors are all known to provide some areas of favorable interaction.

Our study showed that the adsorption of GO with bentonite was better than with kaolinite. Such results may be attributed to differences in clay mineral characteristics. For instance, ref. [48] found an apparent association between clay swelling’s size and the quantity of nanoparticles absorbed. Compared to kaolinite, the structure of bentonite allows for more swelling, which facilitates a greater amount of GO adsorption onto bentonite particles [49]. The K_F_ values (Figure 7 and Figure 8) demonstrated that GO nanoparticle adsorption improves as IS (NaCl or CaCl_2_) increases. According to [27], when the IS of the influent was increased from 0 to 10 mM NaCl, the penetration of GO from soil was only marginally impeded, and considerable retention of GO was seen only at 25 mM NaCl and above. This resulted in relatively high IS GO exhibiting increased mobility; the high movement of GO in the soil is due to GONPs’ comparatively strong negative surface charges. In the end, these results also demonstrate the importance of recognizing and comprehending the decomposition of GO in water sources that contain heterogeneity in clay minerals that can influence the adsorption of GO.

### 2.6. Environmental Implications

Due to their extraordinary properties, GO nanomaterials have been touted as miracle materials with many benefits for agriculture, industries, and environmental remediation. Previous studies reported that GO played a promoting role in plant growth. For instance, ref. [62] found that GO increased the germination rate and the growth of spinach. GO is also utilized as a new fertilizer carrier to supply more nutrients for crops [63]. However, the environmental conditions had a significant effect on GO toxicity. For example, Ref. [64] reported that large amounts of GO accumulated on the roots of cabbage, red spinach, and tomato, promoting reactive oxygen species accumulation and ultimately limiting the growth of these crops. A recent study also suggested that GO increased cadmium uptake by rice in soil [65]. The effect of GO on soil microbial diversity were also investigated [66]. Accordingly, understanding GO nanomaterials behavior in the soil is crucial to regulating their ecological risks. The interaction between clay minerals and GO is critical for identifying GO nanoparticles behavior in natural soil–water ecosystem, which helps in identifying the potential risks of GO. Our findings clearly showed that the chemistry conditions of the solution significantly influence the adsorption of GO nanoparticles onto bentonite and kaolinite, which can supply valuable insight into GO nanoparticles simulation and management in the environment. In this regard, our study clearly indicated that the adsorption of GO nanoparticles on clay minerals enhanced at low pH and high SI (Figure 3 and Figure 4). Soil acidification is likely to increase globally in response to the increase in the global nitrogen deposition in the future [67]. On a global scale, ref. [68] suggested that soil pH reduced by 0.26 units in response to nitrogen deposition. Hence, nitrogen deposition may enhance GO nanoparticles adsorption on clay minerals by reducing the soil pH. Moreover, the acidity induced via nitrogen deposition is neutralized by increasing the dissolution calcium carbonate (CaCO_3_) [69]. The dissolution of CaCO_3_ via increasing acidity leads to Ca^2+^ release, which enhances GO nanoparticle adsorption on clay minerals (Figure 4). Moreover, the area of drylands is expected to expand by 11–23% by the end of this century. This will increase soil pH and salinity, which would affect the adsorption of GO nanoparticles on clay minerals. Taken together, more emphasis should be given to the effects of multiple factor interactions of the solution chemistry conditions and global change factors (e.g., nitrogen deposition, acidification, and aridity) on GO nanoparticle adsorption on clay minerals. Because of the possible decontamination capacities of nano-adsorbents, GO adsorption on both bentonite and kaolinite is essential. The adsorption efficiency and capacity of GO-coated bentonite to remove methylene blue dye from aqueous solutions were both high [70]. In addition, encapsulating iron oxide nanoparticles in kaolin–bentonite composites improved fluoride removal from drinking water, demonstrating the potential for clay–magnetite nanoparticle composites in water treatment [71]. The creation of a chitosan-bentonite-nano-GO nanosorbent also displayed good phenol adsorption, suggesting that polymeric adsorbents might be used to remove aromatic chemicals from polluted water [72]. Overall, investigating GO adsorption on bentonite and kaolinite can help to create effective adsorbents for pollutant removal and environmental remediation.

### 2.7. The Limitations of the Study

The thermodynamic parameters associated with GO adsorption onto clay minerals include the isosteric heat of adsorption, the variation of surface free energy, and the adsorption capacity characteristics. The isosteric heat of adsorption is a measure of the intermolecular forces between the adsorbate and the clay minerals, and it decreases with increasing adsorption capacity [73]. The variation of surface free energy is influenced by pressure and temperature, and it is related to the specific surface area of the clay minerals. The surface free energy increases rapidly with pressure at low pressures and more slowly at higher pressures while it decreases with increasing temperature [74]. The adsorption capacity characteristics of the clay minerals are determined by factors such as surface polarity, interlayer spacing, and specific surface area [75]. These thermodynamic parameters provide insights into the adsorption behavior of GO onto clay minerals and can be used to optimize adsorption processes. The study of thermodynamics reveals the spontaneity and feasibility of the adsorption process [76]. The change in entropy (∆*S*°, J mol^–1^ K), enthalpy (∆*H*°, KJ mol^–1^), and Gibb’s free energy (∆*G*°, KJ mol^–1^) calculate as follows [76]:

(1) $$ln\;K_{c}=\frac{∆S^{{}^{\circ}}}{R}–\frac{∆H^{{}^{\circ}}}{RT}$$

(2) $$K_{c}=\frac{C_{a}}{C_{e}}$$

(3) ∆G°=∆H°−T∆S°

where *K_c_* is the equilibrium constant.

The decrease in values of ∆*G*° with increasing temperature reveals a decrease in the adsorption feasibility at a higher temperature. Moreover, the negative value of ∆*G*° indicates that the adsorption process is spontaneous in nature [76]. Ref. [36] reported that the adsorption capacity of sepiolite clay increased with increasing temperature under the same GO concentration, indicating the importance of the effect of temperature on the adsorption process. This was mainly due to a part of surface bound water and free water was lost from clay at higher temperatures, and the resistance of the adsorption was enhanced, which is ideal for adsorbate molecule diffusion, and ultimately improved the clay mineral adsorption performance [77]. With increasing temperature the specific surface area is increased in response to the increase in the number of exposed broken bonds [78]. This also makes the molecules in GO diffuse faster from the solution to clay [78]. Ref. [37] also showed higher temperatures help to enhance the adsorption capacity of GO on clay minerals mainly due to the enhanced absolute value of standard free energy at higher temperatures. They also suggested that the process of adsorption is an endothermic reaction, which was in line with the results of isotherm fitting. Ref. [79] found that GO adsorption on red sandstone increased with increasing temperature under the same GO concentration. This was mainly due to the enhancement of the activity of GO molecules in the aqueous solution, increasing the possibility of GO particles contacting the adsorption active sites [79]. Ref. [15] found that the K° value was reduced with an elevating temperature, indicating an exothermic attachment process. They also found that the Δ*G*° value increased with elevating temperature, indicating that the adsorption process is not feasible at higher temperatures. We also cannot ignore that a higher operating cost is expected when adsorption occurs at higher temperatures. Furthermore, ref. [80] found a negative value of Δ*H*°, demonstrating that the adsorption process was exothermic. They also noted a negative value of Δ*S*°, indicating that the adsorption process was enthalpy controlled with decreased randomness at the solid/liquid interface. However, the mechanical and thermodynamic properties of GO on kaolinite and bentonite were not included in this study, although they are being developed. Furthermore, the majority of studies used at least seven values of compatibility [16,81]. However, due to the lack of capabilities, our study was limited to only four values of compatibility, which is likely to affect the accuracy of our models. Thus, the use of GO adsorption data in our study to understand the GO nanoparticle behavior in the soil should be interpreted with appropriate caution. Hence, future studies should take into account the mechanical and thermodynamic properties of GO on kaolinite and bentonite using as many compatibility values as possible, which could improve our understanding of the actual status of GO nanoparticle behavior in the soil.

## 3. Materials and Methods

### 3.1. Synthetic of GO Nanoparticles

Graphene oxide is traditionally produced from pure graphite powder using a modified Hummers’ process. According to the Micro Analytical Center at Cairo University’s Automatic Analyzer CHNS (Vario el 111 elemental), the GO product had 472 g kg^–1^ C, 518 g kg^–1^ O, and trace amounts of hydrogen (H) and nitrogen (N). GO nanoparticle stock suspension was made by dissolving 300 mg of GO powder in 1000 mL of distilled water and then sonicating the liquid in a water bath at 100 W for 4 h [21].

### 3.2. Adsorption of GO Nanoparticles on Clay Minerals under Different Chemical Conditions

To determine how the chemistry of solutions impacts GO adsorption on clay particles, the clay minerals were not purified before usage [22,48]. A series of investigations were conducted utilizing GO nanoparticles at 0, 15, 30, 45, and 60 mg GO L^−1^. This experiment included the usage of soil minerals, such as bentonite and kaolinite. Furthermore, GO adsorption on various clay minerals (bentonite and kaolinite) was investigated under various IS of NaCl (10 and 20 mM) and CaCl_2_ (0.1 and 0.3 mM) along with various pH (5.0, 7.0, and 9.0, which adjusted with 10 mM HCl or 10 mM NaOH). The GO was absorbed onto clay particles according to [21]. The adsorption approach was used to make initial concentrations of GO suspensions ranging between 0 and 60 mg GO L^−1^ by adding aliquots of GO stock suspension in various electrolytes in beakers. Then, a 40 mL glass vial was filled with 40 mg of each clay substance. The glass vials have been filled with various levels of GO suspension, leaving only a little head space. The samples were then agitated with a shaker for three days (the period necessary to achieve equilibrium adsorption was specified). Each adsorption experiment was replicated three times. The supernatant was collected after centrifuging the samples at 3500 rpm for 20 min to determine the concentrations of GO in the water solution using a UV-V spectrometer with a wavelength of 230 nm [26]. To calculate the adsorption quantities (*q_e_*; mg GO nanoparticles g^–1^ clay), the isotherm of the adsorption process was created using the following equation:

(4) $$q_{e}=\frac{\left(C_{0}–C_{e}\right)\;V}{m}$$

where *C_0_*, *C_e_*, *V*, and *m* were the GO concentration at initiation, the concentration of GO in the supernatant (equilibrium concentration), the suspension’s volume, and the clay mass, respectively.

The laboratory findings of the constant GO adsorption on clay minerals under various conditions of solution chemistry, which have been used in several colloid adsorption investigations of environmental concern, were fitted using both the Langmuir and Freundlich isotherms [46,57].

The non-linear Langmuir and non-linear Freundlich isotherm models were applied to predict the adsorption capacity of given materials. The non-linear Langmuir equation has been expressed as follows:

(5) $$q_{e}=\frac{Q_{m\times K_{L}\times C_{e}}}{\left[1+K_{L}\times C_{e}\right]}$$

where *C_e_*, *q_e_*, *Q_m_*, and *K_L_* represented the amount of GO in the equilibrium solution (mg L^−1^), the amount of GO adsorbed per mass unit of soil (mg kg^−1^), the maximum adsorption (mg kg^−1^), and the bonding energy constant (L mg^−1^).

The Langmuir adsorption maxima and bonding energy constant were calculated by regressing *C_e_*/*q_e_* against *C_e_* and obtaining the slope and intercept of the stated relationship, respectively. Maximum adsorption is the reciprocal of the slope, and the constant of bonding energy is the reciprocal of the intercept to the adsorption maxima. The Langmuir coefficients and the Chi-square (*x*^2^) for the equation of non-linearized Langmuir were obtained by plotting graphs between *q_e_* vs. *C_e_*. The non-linear Freundlich equation has been expressed as follows [82]:

(6) $$q_{e}=K_{F}C_{e}^{1/n}$$

where *C_e_* was the GO concentration in the equilibrium solution (mg L^−1^), *q_e_* was the quantity of GO adsorbed per mass unit of soil (mg kg^−1^), *K_F_* was the measure of adsorptive capacity (mg kg^−1^), and *n* was the intensity of adsorption (L kg^−1^). According to [83], the 1/*n* shows the adsorption isotherm’s divergence from linearity. By regressing q_e_ against *C_e_*, it was possible to determine the Freundlich adsorptive capacity and adsorption intensity, or sorption energy constant, from the slope and intercept of the relationship, respectively. Adsorption energy or intensity was inversely proportional to slope while intercept (*K_F_*) had an inverse relationship with the adsorptive capacity constant. The capacity for attachment of the sorbent was directly correlated with *K_F_*, and the surface heterogeneity of the sorbent was characterized by 1/*n* [46]. The 1/*n* values equal one indicated that the isotherm was linear while the 1/*n* values lower than one indicated that adsorption was favorable convex, and the opposite was recorded with the 1/*n* values higher than one. The parameters of the associated Freundlich isotherm were determined. The Freundlich coefficients and the Chi-square (*x*^2^) for the equation of non-linearized Freundlich were obtained by plotting graphs between *q_e_* vs. *C_e_*.

## 4. Conclusions

The findings of this experiment demonstrated how GO interacts with distinct clay minerals. The batch investigations revealed that the solution chemistry conditions had a substantial impact on the adsorption of GO nanoparticles onto clay minerals. As the ionic force rises, there is a noticeable rise in adsorption when GO nanoparticles are added to clay minerals. This may be related to electrostatic relations among GO and clay minerals. In addition, when increasing the pH from 5.0 to 9.0, the amount of GO nanoparticles that attached to clay minerals’ surfaces reduced. This is related to electrostatic forces and the clay mineral framework. The adsorption of GO may be significantly inhibited due to increased negative charges by adsorbed organic ligands and competition for binding sites by ligands with GO surface groups. These results highlight the importance of detecting and comprehending the fate of GO in natural soil–water ecosystems, which include different clay minerals that could influence GO adsorption.

## Data Availability

The data that support the findings of this study are available from the corresponding author upon reasonable request.

## Sample Availability

Samples of the compounds are not available from the authors.

## References

[1] Shen C., Lazouskaya V., Zhang H., Li B., Jin Y., Huang Y. (2013). Influence of surface chemical heterogeneity on attachment and detachment of microparticles. Colloids Surf. A Physicochem. Eng. Asp..

[2] Bhattacharya M. (2016). Polymer nanocomposites—A comparison between carbon nanotubes, graphene, and clay as nanofillers. Materials.

[3] Yang L., Zhang R., Liu B., Wang J., Wang S., Han M.Y., Zhang Z. (2014). Π-conjugated carbon radicals at graphene oxide to initiate ultrastrong chemiluminescence. Angew. Chem. Int. Ed..

[4] Compton O.C., Nguyen S.T. (2010). Graphene oxide, highly reduced graphene oxide, and graphene: Versatile building blocks for carbon-based materials. Small.

[5] Wang X., Chen L., Wang L., Fan Q., Pan D., Li J., Chi F., Xie Y., Yu S., Xiao C. (2019). Synthesis of novel nanomaterials and their application in efficient removal of radionuclides. Sci. China Chem..

[6] Kong Q., Preis S., Li L., Luo P., Wei C., Li Z., Hu Y., Wei C. (2020). Relations between metal ion characteristics and adsorption performance of graphene oxide: A comprehensive experimental and theoretical study. Sep. Purif. Technol..

[7] Wang X., Liu Y., Pang H., Yu S., Ai Y., Ma X., Song G., Hayat T., Alsaedi A., Wang X. (2018). Effect of graphene oxide surface modification on the elimination of Co (II) from aqueous solutions. Chem. Eng. J..

[8] He J.-Z., Li Z.-Z., Wang D.-J., Zhou D.-M. (2015). Biofilms and extracellular polymeric substances mediate the transport of graphene oxide nanoparticles in saturated porous media. J. Hazard. Mater..

[9] Mazive P.A., Hu B., Zhu H., He W., Mazive A.M. (2020). Graphene Oxide-Based Nanomaterials: The Preparation, Application, and Factors that Affect the Adsorption Capacity on Drinking Water Treatment-Review. J. Nanotechnol. Res..

[10] Lanphere J.D., Rogers B., Luth C., Bolster C.H., Walker S.L. (2014). Stability and transport of graphene oxide nanoparticles in groundwater and surface water. Environ. Eng. Sci..

[11] Zhou D., Jiang X., Lu Y., Fan W., Huo M., Crittenden J. (2016). Cotransport of graphene oxide and Cu (II) through saturated porous media. Sci. Total Environ..

[12] Wang D., Jin Y., Jaisi D.P. (2015). Cotransport of hydroxyapatite nanoparticles and hematite colloids in saturated porous media: Mechanistic insights from mathematical modeling and phosphate oxygen isotope fractionation. J. Contam. Hydrol..

[13] Wang H., Dong Y.-n., Zhu M., Li X., Keller A.A., Wang T., Li F. (2015). Heteroaggregation of engineered nanoparticles and kaolin clays in aqueous environments. Water Res..

[14] Marouf R., Dali N., Boudouara N., Ouadjenia F., Zahaf F. (2021). Study of adsorption properties of bentonite clay. Montmorillonite Clay.

[15] Sotirelis N.P., Chrysikopoulos C.V. (2017). Heteroaggregation of graphene oxide nanoparticles and kaolinite colloids. Sci. Total Environ..

[16] Song X., Zhou L., Kang H., Li N., Wang W., Jiang P. (2022). Study on Adsorption Properties and Mechanism of Graphene Oxide (GO) by Kaolin. Nat. Environ. Pollut. Technol..

[17] Puri C., Sumana G. (2018). Highly effective adsorption of crystal violet dye from contaminated water using graphene oxide intercalated montmorillonite nanocomposite. Appl. Clay Sci..

[18] Bao T., Damtie M.M., Wu K., Wei X.L., Zhang Y., Chen J., Deng C.X., Jin J., Yu Z.M., Wang L. (2019). Rectorite-supported nano-Fe_3_O_4_ composite materials as catalyst for P-chlorophenol degradation: Preparation, characterization, and mechanism. Appl. Clay Sci..

[19] Mouni L., Belkhiri L., Bollinger J.-C., Bouzaza A., Assadi A., Tirri A., Dahmoune F., Madani K., Remini H. (2018). Removal of Methylene Blue from aqueous solutions by adsorption on Kaolin: Kinetic and equilibrium studies. Appl. Clay Sci..

[20] He G., Wang C., Cao J., Fan L., Zhao S., Chai Y. (2019). Carboxymethyl chitosan-kaolinite composite hydrogel for efficient copper ions trapping. J. Environ. Chem. Eng..

[21] Lu X., Lu T., Zhang H., Shang Z., Chen J., Wang Y., Li D., Zhou Y., Qi Z. (2019). Effects of solution chemistry on the attachment of graphene oxide onto clay minerals. Environ. Sci. Process. Impacts.

[22] Lu T., Xia T., Qi Y., Zhang C., Chen W. (2017). Effects of clay minerals on transport of graphene oxide in saturated porous media. Environ. Toxicol. Chem..

[23] Syngouna V.I., Chrysikopoulos C.V. (2010). Interaction between viruses and clays in static and dynamic batch systems. Environ. Sci. Technol..

[24] Chrysikopoulos C.V., Sotirelis N.P., Kallithrakas-Kontos N.G. (2017). Cotransport of graphene oxide nanoparticles and kaolinite colloids in porous media. Transp. Porous Media.

[25] Amar A., Loulidi I., Kali A., Boukhlifi F., Hadey C., Jabri M. (2021). Physicochemical Characterization of Regional Clay: Application to Phenol Adsorption. Appl. Environ. Soil Sci..

[26] Qi Z., Zhang L., Wang F., Hou L., Chen W. (2014). Factors controlling transport of graphene oxide nanoparticles in saturated sand columns. Environ. Toxicol. Chem..

[27] Qi Z., Zhang L., Chen W. (2014). Transport of graphene oxide nanoparticles in saturated sandy soil. Environ. Sci. Process. Impacts.

[28] Zhang L., Hou L., Wang L., Kan A.T., Chen W., Tomson M.B. (2012). Transport of fullerene nanoparticles (n C60) in saturated sand and sandy soil: Controlling factors and modeling. Environ. Sci. Technol..

[29] Ryan J.N., Elimelech M. (1996). Colloid mobilization and transport in groundwater. Colloids Surf. A Physicochem. Eng. Asp..

[30] Feng Y., Huynh K.A., Xie Z., Liu G., Gao S. (2019). Heteroaggregation and sedimentation of graphene oxide with hematite colloids: Influence of water constituents and impact on tetracycline adsorption. Sci. Total Environ..

[31] Tetteh S., Zugle R., Ofori A., Adotey J.P.K. (2020). Kinetics and equilibrium thermodynamic studies of the adsorption of phenolphthalein and methyl orange onto muscovite clay. Front. Chem. Res..

[32] Olaofe O., Olagboye S., Akanji P., Adamolugbe E., Fowowe O., Olaniyi A. (2015). Kinetic studies of adsorption of heavy metals on clays. Int. J. Chem..

[33] Syngouna V.I., Giannadakis G.I., Chrysikopoulos C.V. (2020). Interaction of graphene oxide nanoparticles with quartz sand and montmorillonite colloids. Environ. Technol..

[34] Nas M.S. (2019). The investigation of thermodynamics parameters and adsorption kinetic of the maxilon blue 5G dye on Turkey green clay. J. Inst. Sci. Technol..

[35] Ahmat A.M., Thiebault T., Guégan R. (2019). Phenolic acids interactions with clay minerals: A spotlight on the adsorption mechanisms of Gallic Acid onto montmorillonite. Appl. Clay Sci..

[36] Li N., Yan X., Dai W., Lv B., Wang W. (2023). Adsorption properties and mechanism of sepiolite to graphene oxide in aqueous solution. Arab. J. Chem..

[37] Li N., Fang J., Jiang P., Li C., Kang H., Wang W. (2022). Adsorption properties and mechanism of attapulgite to graphene oxide in aqueous solution. Int. J. Environ. Res. Public Health.

[38] Zhao J., Liu F., Wang Z., Cao X., Xing B. (2015). Heteroaggregation of graphene oxide with minerals in aqueous phase. Environ. Sci. Technol..

[39] Wang M., Zhang H., Chen W., Lu T., Yang H., Wang X., Lu M., Qi Z., Li D. (2021). Graphene oxide nanoparticles and hematite colloids behave oppositely in their co-transport in saturated porous media. Chemosphere.

[40] Xia T., Lin Y., Guo X., Li S., Cui J., Ping H., Zhang J., Zhong R., Du L., Han C. (2019). Co-transport of graphene oxide and titanium dioxide nanoparticles in saturated quartz sand: Influences of solution pH and metal ions. Environ. Pollut..

[41] Adam V., Loyaux-Lawniczak S., Labille J., Galindo C., Del Nero M., Gangloff S., Weber T., Quaranta G. (2016). Aggregation behaviour of TiO_2_ nanoparticles in natural river water. J. Nanopart. Res..

[42] Dong F., Zhou Y. (2020). Distinct mechanisms in the heteroaggregation of silver nanoparticles with mineral and microbial colloids. Water Res..

[43] Guo Q., Wang Z., Xu Q., Mao H., Zhang D., Ghosh S., Pradhan N.R., Pan B., Xing B. (2020). Suspended state heteroaggregation kinetics of kaolinite and fullerene (nC60) in the presence of tannic acid: Effect of π-π interactions. Sci. Total Environ..

[44] Li X., He E., Zhang M., Peijnenburg W.J., Liu Y., Song L., Cao X., Zhao L., Qiu H. (2020). Interactions of CeO_2_ nanoparticles with natural colloids and electrolytes impact their aggregation kinetics and colloidal stability. J. Hazard. Mater..

[45] Johnston C.T., Tombacz E. (2002). Surface chemistry of soil minerals. Soil Miner. Environ. Appl..

[46] Sotirelis N.P., Chrysikopoulos C.V. (2015). Interaction between graphene oxide nanoparticles and quartz sand. Environ. Sci. Technol..

[47] Bradford S.A., Torkzaban S. (2012). Colloid adhesive parameters for chemically heterogeneous porous media. Langmuir.

[48] Omurlu C., Pham H., Nguyen Q. (2016). Interaction of surface-modified silica nanoparticles with clay minerals. Appl. Nanosci..

[49] Angioi S., Polati S., Roz M., Rinaudo C., Gianotti V., Gennaro M. (2005). Sorption studies of chloroanilines on kaolinite and montmorillonite. Environ. Pollut..

[50] Wang H., Adeleye A.S., Huang Y., Li F., Keller A.A. (2015). Heteroaggregation of nanoparticles with biocolloids and geocolloids. Adv. Colloid Interface Sci..

[51] Kaya A., Yukselen Y. (2005). Zeta potential of clay minerals and quartz contaminated by heavy metals. Can. Geotech. J..

[52] Song L., Johnson P.R., Elimelech M. (1994). Kinetics of colloid deposition onto heterogeneously charged surfaces in porous media. Environ. Sci. Technol..

[53] Chen J.Y., Ko C.-H., Bhattacharjee S., Elimelech M. (2001). Role of spatial distribution of porous medium surface charge heterogeneity in colloid transport. Colloids Surf. A Physicochem. Eng. Asp..

[54] Tian Y., Gao B., Wu L., Muñoz-Carpena R., Huang Q. (2012). Effect of solution chemistry on multi-walled carbon nanotube deposition and mobilization in clean porous media. J. Hazard. Mater..

[55] Shih C.-J., Wang Q.H., Lin S., Park K.-C., Jin Z., Strano M.S., Blankschtein D. (2012). Breakdown in the wetting transparency of graphene. Phys. Rev. Lett..

[56] Idris O.A., Ahmed H.S. (2012). Phosphorus sorption capacity as a guide for phosphorus availability of selected Sudanese soil series. Afr. Crop Sci. J..

[57] Chrysikopoulos C.V., Aravantinou A.F. (2014). Virus attachment onto quartz sand: Role of grain size and temperature. J. Environ. Chem. Eng..

[58] Chrysikopoulos C.V., Syngouna V.I. (2012). Attachment of bacteriophages MS2 and ΦX174 onto kaolinite and montmorillonite: Extended-DLVO interactions. Colloids Surf. B Biointerfaces.

[59] Subramanyam B., Das A. (2009). Linearized and non-linearized isotherm models comparative study on adsorption of aqueous phenol solution in soil. Int. J. Environ. Sci. Technol..

[60] Tombácz E., Szekeres M. (2006). Surface charge heterogeneity of kaolinite in aqueous suspension in comparison with montmorillonite. Appl. Clay Sci..

[61] Zhou D., Abdel-Fattah A.I., Keller A.A. (2012). Clay particles destabilize engineered nanoparticles in aqueous environments. Environ. Sci. Technol..

[62] He Y., Hu R., Zhong Y., Zhao X., Chen Q., Zhu H. (2018). Graphene oxide as a water transporter promoting germination of plants in soil. Nano Res..

[63] Kabiri S., Degryse F., Tran D.N., da Silva R.C., McLaughlin M.J., Losic D. (2017). Graphene oxide: A new carrier for slow release of plant micronutrients. ACS Appl. Mater. Interfaces.

[64] Begum P., Ikhtiari R., Fugetsu B. (2011). Graphene phytotoxicity in the seedling stage of cabbage, tomato, red spinach, and lettuce. Carbon.

[65] He Y., Qian L., Zhou K., Hu R., Huang M., Wang M., Zhao G., Liu Y., Xu Z., Zhu H. (2019). Graphene oxide promoted cadmium uptake by rice in soil. ACS Sustain. Chem..

[66] Forstner C., Orton T.G., Skarshewski A., Wang P., Kopittke P.M., Dennis P.G. (2019). Effects of graphene oxide and graphite on soil bacterial and fungal diversity. Sci. Total Environ..

[67] Reay D.S., Dentener F., Smith P., Grace J., Feely R.A. (2008). Global nitrogen deposition and carbon sinks. Nat. Geosci..

[68] Tian D., Niu S. (2015). A global analysis of soil acidification caused by nitrogen addition. Environ. Res. Lett..

[69] Elrys A.S., Raza S., Elnahal A.S., Na M., Ahmed M., Zhou J., Chen Z. (2020). Do soil property variations affect dicyandiamide efficiency in inhibiting nitrification and minimizing carbon dioxide emissions?. Ecotoxicol. Environ. Saf..

[70] Shaarawy H., Hussein H., Kader E.A., Hussien N.H., Hawash S. (2020). Adsorption performance of coated bentonite via graphene oxide. Bull. Natl. Res. Cent..

[71] Annan E., Nyankson E., Agyei-Tuffour B., Armah S.K., Nkrumah-Buandoh G., Hodasi J.A.M., Oteng-Peprah M. (2021). Synthesis and characterization of modified kaolin-bentonite composites for enhanced fluoride removal from drinking water. Adv. Mater. Sci. Eng..

[72] Jadid A., Shahsavari S., Seifkordi A., Yazdi A.V. (2021). Adsorption of Phenol in Wastewater Using Nano Grapheme Oxide-Chitosan-Bentonite Absorbent. Depiction Health.

[73] Ismadji S., Soetaredjo F.E., Ayucitra A., Ismadji S., Soetaredjo F.E., Ayucitra A. (2015). The equilibrium studies in the adsorption of hazardous substances using clay minerals. Clay Materials for Environmental Remediation.

[74] Liang L., Xiong J., Liu X., Luo D. (2016). An investigation into the thermodynamic characteristics of methane adsorption on different clay minerals. J. Nat. Gas Sci. Eng..

[75] He H., Zhu J. (2017). Analysis of organoclays and organic adsorption by clay minerals. Developments in Clay Science.

[76] Zafar S., Khan M.I., Rehman H.U., Fernandez-Garcia J., Shahida S., Prapamonthon P., Khraisheh M., Rehman A.U., Ahmad H.B., Mirza M.L. (2020). Kinetic, equilibrium, and thermodynamic studies for adsorptive removal of cobalt ions by rice husk from aqueous solution. Desalination Water Treat..

[77] Raya I., Widjaja G., Mahmood Z.H., Kadhim A.J., Vladimirovich K.O., Mustafa Y.F., Kadhim M.M., Mahmudiono T., Husein I., Kafi-Ahmadi L. (2022). Kinetic, isotherm, and thermodynamic studies on Cr (VI) adsorption using cellulose acetate/graphene oxide composite nanofibers. Appl. Phys. A.

[78] Foo K., Hameed B. (2013). Utilization of oil palm biodiesel solid residue as renewable sources for preparation of granular activated carbon by microwave induced KOH activation. Bioresour. Technol..

[79] Li N., Dai W., Kang H., Lv B., Jiang P., Wang W. (2022). Study on the adsorption performance and adsorption mechanism of graphene oxide by red sandstone in aqueous solution. Adsorpt. Sci. Technol..

[80] Deepthi Rani R., Sasidhar P. (2012). Sorption of cesium on clay colloids: Kinetic and thermodynamic studies. Aquat. Geochem..

[81] Jahan N., Roy H., Reaz A.H., Arshi S., Rahman E., Firoz S.H., Islam M.S. (2022). A comparative study on sorption behavior of graphene oxide and reduced graphene oxide towards methylene blue. Case Stud. Chem. Environ. Eng..

[82] Freundlich H. (1926). New conception in colloidal chemistry, colloid and capillary chemistry. Methuen.

[83] Isa M.H., Lang L.S., Asaari F.A., Aziz H.A., Ramli N.A., Dhas J.P.A. (2007). Low cost removal of disperse dyes from aqueous solution using palm ash. Dye Pigment..

